# Hospital pharmacy workforce in Brazil

**DOI:** 10.1186/s12960-017-0265-5

**Published:** 2018-01-04

**Authors:** Thiago R. Santos, Jonathan Penm, André O. Baldoni, Lorena Rocha Ayres, Rebekah Moles, Cristina Sanches

**Affiliations:** 1 Federal University of Sao Joao del Rei, Campus Centro-Oeste Dona Lindu, Av. Sebastião Gonçalves Coelho, 400 – Chanadour, Divinópolis, MG CEP 35.501-296 Brazil; 2 Hospital Pharmacy Section, International Pharmaceutical Federation, The Hague, The Netherlands; 3 Faculty of Pharmacy, University of Sydney, Camperdown, NSW Australia; 4 Department of Pharmaceutical Sciences, Federal University of Espírito Santo, Vitória, Brazil

**Keywords:** Health services, Hospital pharmaceutical service, Pharmacists, Brazil

## Abstract

**Background:**

This study aims to describe the distribution of the hospital pharmacy workforce in Brazil.

**Methods:**

Data were acquired, during 2016, through the Brazilian National Database of Healthcare Facilities (CNES). The following variables were extracted: hospital name, registry number, telephone, e-mail, state, type of institution, subtype, management nature, ownership, presence of research/teaching activities, complexity level, number of hospital beds, presence of pharmacists, number of pharmacists, pharmacist specialization. All statistical analyses were performed by IBM SPSS v.19.

**Results:**

The number of hospitals with a complete registry in the national database was 4790. The majority were general hospitals (77.9%), managed by municipalities (66.1%), under public administration (44.0%), had no research/teaching activities (90.5%), classified as medium complexity (71.6%), and had no pharmacist in their team (50.6%). Furthermore, almost 60.0% of hospitals did not comply with the minimum recommendations of having a pharmacist per 50 hospital beds. The Southeast region had the highest prevalence of pharmacists, with 64.4% of hospitals having a pharmaceutical professional. This may have occurred as this region had the highest population to hospital ratio. Non-profit hospitals were more likely to have pharmacists compared to those under public administration and private hospitals.

**Conclusion:**

This study mapped the hospital pharmacy workforce in Brazil, showing a higher prevalence of hospital pharmacists in the Southeast region, and in non-profit specialized hospitals.

## Background

The third Global Patient Safety Challenge is Medication Safety. This highlights the importance of trying to diminish unsafe medication practices and medication errors. The impact of these errors is particularly evident in the hospital setting. Key policy documents highlight the skills of the pharmacists to conduct medication reconciliation and to reduce polypharmacy [1]. In 2013, the World Health Organization (WHO) estimated a global workforce shortage of 7.2 million healthcare professionals [2]. As such, WHO recommends that each country develop a national health workforce strategy that prepares the future workforce, enhances current worker performances, and manages attrition [3]. This strategy should help prepare the workforce response to the aging population and growing burden of chronic disease [4, 5].

Pharmacists’ expertise is particularly important in the hospital setting where patients are acutely unwell and often started on invasive, high-risk medications that can interact with other pre-existing medications. As such, the International Pharmaceutical Federation released the Basel Statements for the future of hospital pharmacy that state: “Health authorities should ensure that each hospital is serviced by a pharmacy that is supervised by pharmacists who have completed advanced training in hospital pharmacy” [6, 7].

Furthermore, the Basel Statements also target human resources, training and development issues and state:

Hospitals should maintain human resource information systems that contain basic data for planning, training, appraising, and supporting the workforce. Data should be collated at a national level to improve workforce planning.

The Basel Statements further highlight the important role of hospital pharmacists to optimize patient outcomes through responsible use of medicines. This includes not only timely access to safe and effective medicines, but that medicines are only used when necessary and that the choice of medicine is appropriate based on clinical evidence and safety to prevent medication errors [8–11]. To ensure all patients receive appropriate medications, some countries like the United States of America (USA) have also utilized pharmacy support staff, such as pharmacy technicians, to take on technical and basic clinical responsibilities while their pharmacists target advanced care patients [12].

Brazil has also acknowledged the importance of hospital pharmacists with laws stating they must be present in all hospitals [13, 32]. In addition, the Minimum Standards for Hospital Pharmacy and Healthcare Services recommend at least one pharmacist for every 50 hospital beds for basic dispensing services [14]. Despite these recommendations and growing evidence that hospital pharmacy services improve patients’ clinical outcomes [15, 16], low compliance with these legal requirements and standards have been observed [17]. Brazil currently has a higher density of pharmacists (9.1 per 10,000 population) than the global average (6 per 10,000 population) with growing capacity over the past years [4, 18]. Additionally, expressive gains were obtained concerning pharmaceutical care [19–21]. However, to the best of our knowledge, current studies have not evaluated the distribution of the hospital pharmacy workforce in Brazil [4, 18]. This study aimed to describe the pharmacy workforce distribution in hospital settings in Brazil.

## Methods

This was a cross-sectional study.

Data were acquired through the National Database of Healthcare Facilities (CNES), via website (http://cnes2.datasus.gov.br). The following variables hospital name, registry number, telephone, e-mail, address, state, type and subtype of institution, management nature, ownership, presence of research/teaching activities, hospital complexity level, number of hospital beds, presence of pharmacists, number of pharmacists, and pharmacist specialization were extracted, aided by an online platform provided by Google Forms. Data were collected from a single government-run database from February of 2016 to September of 2016.

States were coded and clustered into regions, as defined by IBGE - North, Northeast, Central-West, Southeast and South. Management was divided into city, state or both. Hospitals with both city and state management were labeled as “joint” [22].

According to Brazilian classification, medium complexity hospitals are those with availability of specialized professionals and technological resources aimed at resolving major health issues of the local population, and high complexity hospitals are those with high technology resources and costs, aiming to provide qualified services to the population, integrating those services with the other levels of health assistance, as primary care and medium complexity [23].

Legal ownership was also clustered into three categories as defined by IBGE, which are (1) Public; (2) Private, and (3) Non-profit organization, as shown in Table 1.

**Table 1:** Legal ownership clustering, as defined by IBGE [43]

Classification	Types
Public	City
	City foundation
	Public agency of municipality executive power
	Public agency of federal executive power
	Public agency of state or federal district executive power
	State or federal district
	State or federal district autarchy
	State or federal district foundation
	Public foundation of municipality private law
	City autarchy
	Federal autarchy
	Public association
Private	Limited company
	Limited liability company (of simple nature)
	Limited partnership
	Closed corporation
	Pure simple society
	Cooperative company
	Businessman (individual)
	Simple society in collective name
	Limited liability company (of business nature)
	Open corporation
	Joint capital business
	Public corporation
Non-profit	Private association
	Private foundation
	Autonomous social service
	Syndical entity
	Social organization
	Religious organization

Medical laboratory scientists were not counted as hospital pharmacists despite some crossover in roles in the country. Additionally, pharmacy technicians are not registered at CNES; therefore, data regarding these support staff were not collected.

Maps were produced by Google Maps online system, using hospitals’ names and addresses. Absence of pharmacists was considered when no registry of pharmacists working for hospitals was found.

Statistical analyses were performed using IBM SPSS 19.0. The presence or absence of hospital pharmacists was compared with the following variables: type of institution, presence of research/teaching activities, complexity, region, subtype, management nature, and ownership. The continuous variable, number of hospitals beds, was recodified to a nominal variable according the median value of 40 hospital beds. Data normality was evaluated by Kolmogorov-Smirnorv test; medians of number of hospital beds were compared by Mann-Whitney *U* test. Nominal and categorical data were compared by binary logistic regression (odds ratio and 95%CI). Variables with *p* < 0.2 from the univariate analysis and those with plausibility to be inserted according to the literature were included in the multivariate model. The variable “subtypes of specialized hospital” was not included, as it is a subpart of “type of institution”. The stepwise backward method was used in order to insert the variables, and only the significant variables with *p* < 0.5 remained. The explanatory variables used in the multivariate model were as follows: type of institution, presence of research/teaching activities, complexity, region, management nature, and ownership. Data were considered statistically significant when *p* < 0.05.

States’ Gross Domestic Product (GDP) obtained at IBGE [22] was correlated to the total number of pharmaceutical professionals in each of the 27 Brazilian States, by Spearman correlation.

## Results

**Fig. 1:**
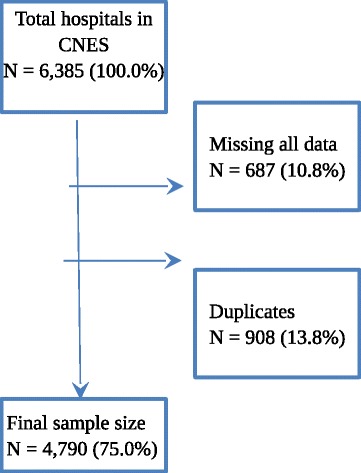
Selection flowchart of hospitals for data analysis

From a total of 6385 registries of hospitals in the National Database of Healthcare Facilities, 908 (13.8%) were duplicates and 687 (10.8%) were missing all data. Hence, these were excluded. The final sample size was 4790 (75.0%) (Fig. 1).

The distribution of hospitals among the Brazilian regions is presented in Table 2. The North region had the least number of hospitals and the Northeast region had the highest number of hospitals. Regarding the number of hospital beds per 1000 people, the lowest and highest ratios were the North and South regions respectively.

**Table 2:** Hospital distribution among Brazilian geographic regions compared with population distribution and hospital beds [44]

Region	Frequency *n* (%)	Hospital beds *n* (%)	Region population [44]	Hospital beds/10 000 population
Central-West	604 (12.6)	26 173 (8.4)	15 219 608	17.2
North	423 (8.8)	23 315 (7.5)	17 231 027	13.5
Northeast	1 539 (32.1)	83 404 (26.8)	56 186 190	14.8
South	746 (15.6)	51 132 (16.4)	29 016 144	17.6
Southeast	1 478 (30.9)	127 091 (40.8)	85 115 623	14.9
Total	4 790 (100)	311 115 (100)	202 768 562	15.3

Hospital characteristics are described in Table 3.

**Table 3:** Description of Brazilian hospitals considering type, subtypes, management nature, ownership, teaching, and complexity

	Absolute frequency (*n*)	Relative frequency (%)
Type of institution		
General hospital	3 732	77.9
Specialized hospital	1 058	22.1
Total	4 790	100.0
Subtypes of specialized hospitals		
Cardiology	64	6.1
Maternity	154	14.6
Oncology	56	5.3
Orthopaedics	47	4.4
Paediatrics	114	10.8
Psychiatry	230	21.7
NA	393	37.1
Total	1 058	100.0
Management nature		
Municipality	3 168	66.1
Joint	906	18.9
State	716	14.9
Total	4 790	100.0
Legal ownership		
Private	1 511	31.5
Non-profit	1 170	24.4
Public	2 109	44.0
Total	4 790	100.0
Presence of teaching/research activity		
Yes	457	9.5
No	4 333	90.5
Total	4 790	100.0
Complexity level		
High complexity	1 359	28.4
Medium complexity	3 428	71.6
NA	3	0.9
Total	4 790	100.0

*NA* information not available

Regarding the presence of pharmacists, more than half of the hospitals lacked a hospital pharmacist in their healthcare team, being 2426 (50.6%) institutions without a single pharmacist versus 2364 (49.4%) with one or more pharmacists (Table 4). There was a significant association between number of hospital beds and the presence of pharmacists. Hospitals with a median of 30 (15–50, interquartile range) hospital beds were those with the absence of the professional, while a median of 58 (32–116) beds had at least one pharmacist (*p* < 0.0001).

**Table 4:** Brazilian hospital characteristics and the factors associated with the presence of the pharmacist, *n* = 4 790, 2016

Variable		Presence of pharmacist		Univariate analysis			Multivariate analysis		
		No (*n* = 2 426)	Yes (*n* = 2 364)	OR	IC95%	*p* value	OR	IC95%	*p* value
Type of institution	General hospital	1 921 (51.5%)	1 811 (48.5%)	1			1		
	Specialized hospital	505 (47.7%)	553 (52.3%)	1.16	1.01–1.33	0.032	1.35	1.14–1.61	<0.0001
Subtypes of specialized hospital	Orthopaedics	26 (55.3%)	21 (44.7%)	1					
	Oncology	9 (16.1%)	47 (83.9%)	6.47	2.59–16.16	< 0.0001			
	Paediatrics	53 (46.5%)	61 (53.5%)	1.42	0.72–2.82	0.309			
	Maternity	50 (32.5%)	104 (67.5%)	2.57	1.32–5.02	0.005			
	Psychiatry	70 (30.4%)	160 (69.6%)	2.83	1.49–5.37	0.001			
	Cardiology	29 (45.3%)	35 (54.7%)	1.49	0.70–3.18	0.298			
	NA	268 (68.2%)	125 (31.8%)						
Management nature	Municipality	1 671 (52.7%)	1 497 (47.3%)	1			1		
	Joint	513 (56.6%)	393 (43.4%)	0.86	0.74–0.99	0.039	0.85	0.71–1.02	0.089
	State	242 (33.8%)	474 (66.2%)	2.19	1.84–2.59	< 0.0001	1.56	1.28–1.89	< 0.0001
Legal ownership	Public	1 012 (48.0%)	1 097 (52.0%)	1			1		
	Non-profit	424 (36.2%)	746 (63.8%)	1.62	1.40–1.88	< 0.0001	0.837	0.70–1.00	0.045
	Private	990 (65.5%)	521 (34.5%)	0.48	0.42–0.56	< 0.0001	0.32	0.27–0.38	< 0.0001
Presence of teaching/research activity	No	2 341 (54.0%)	1 992 (46.0%)	1			1		
	Yes	85 (18.6%)	372 (81.4%)	5.14	4.03–6.56	< 0.0001	1.91	1.45–2.51	< 0.0001
Complexity level*	Medium complexity	1 980 (57.8%)	1 448 (42.2%)	1			1		
	High complexity	443 (32.6%)	916 (67.4%)	2.83	2.48–3.23	< 0.0001	1.92	1.64–2.26	< 0.0001
Region	Northeast Region	956 (62.1%)	583 (37.9%)	1			1		
	North Region	258 (60.9%)	165 (39.1%)	1.05	0.84–1.31	0.673	0.96	0.75–1.21	0.720
	South Region	320 (42.9%)	426 (57.1%)	2.18	1.83–2.61	< 0.0001	2.23	1.81–2.74	< 0.0001
	Central-West Region	366 (60.6%)	238 (39.4%)	1.07	0.88–1.29	0.514	1.39	1.12–1.72	0.003
	Southeast Region	526 (35.6%)	952 (64.4%)	2.97	2.56–3.44	< 0.0001	2.76	2.2–3.22	< 0.0001
Hospital beds	< 40 beds	1562 (66.8%)	778 (33.2%)	1			1		
	≥ 40 beds	864 (35.3%)	1586 (64.7%)	3.685	3.271–4.153	< 0.0001	2.51	2.19–2.87	< 0.0001

Data expressed as *n* (%), logistic regression

*NA* not available

*n = 4787

**Fig. 2:**
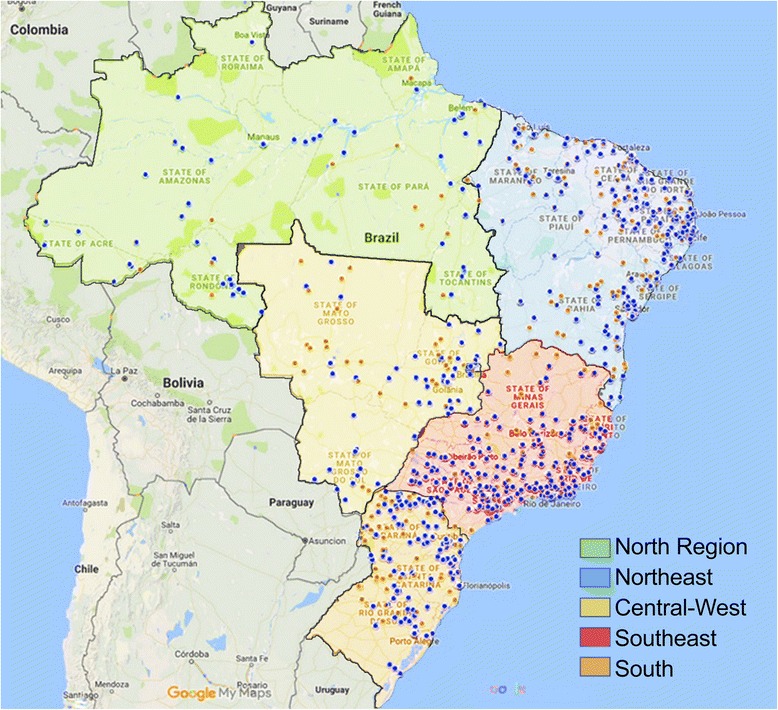
Geographic localization of Brazilian hospitals with pharmacists (blue circles) and without pharmacists (orange circles), according CNES (Google Maps®)

Figure 2 presents the geographic distribution of pharmacists. From 2438 hospitals with a pharmacist, 707 (29.0%) did not meet the minimum national requirement of one pharmacist per 50 hospital beds [18]. Ninety (3.7%) hospitals did not contain enough information to calculate a pharmacist to hospital beds ratio.

A positive correlation was obtained between GDP and the total number of pharmaceutical professionals in each of the 27 Brazilian states (*r*^2^ 0.958; *p* < 0.0001).

## Discussion

Worldwide, the role of hospital pharmacists in promoting responsible use of medicines has made them an invaluable resource [8, 9]. However, according to the main findings of our study, pharmacists were present in less than half of the Brazilian hospitals and were unevenly distributed across the country. These findings reinforce the urgent need to implement policies and procedures to track and monitor the presence of pharmacists in hospitals, practicing both clinically and administratively.

Even though the presence of pharmacists is assured by law in Brazil [13, 32], more than half (50.9%) of the hospitals lacked a pharmaceutical professional on their team. The Minimum Standards for Hospital Pharmacy and Healthcare Services [14] recommend at least one pharmacist per 50 hospital beds. Almost 60% of the hospitals did not comply with this recommendation, working with critically low human resources. Far beyond the legal implications [22], the presence of hospital pharmacists is vital in clinical settings, avoiding clinical errors and improving quality of care [24–28]. Furthermore, the Brazilian Pharmacy Council recognizes six core activities of hospital pharmacy, including the following: 1. Management; 2. Infrastructure development; 3. Preparation, distribution, dispensing and control of healthcare products; 4. Optimization of drug therapy; 5. Provision of information about drugs and healthcare products; and 6. Providing education and research [29]. The absence of this particular professional therefore impairs the quality of the health care services provided by these medical facilities.

Additionally, the Basel Statements for the future of hospital pharmacy, produced under the auspice of FIP also states six core areas of hospital pharmacist activities that include pharmacists overseeing procurement; influences on prescribing; preparation and delivery of medicines; administration; monitoring of medicine use; and Human Resources and training [4–7]. These global statements therefore align with those of the Brazilian Pharmacy Council and highlight the positive role that pharmacists can make to improve the health care service and patient outcomes.

In this study, the presence of pharmacists was higher in hospitals with Research/Teaching Activities. Even so, 18.6% of them still did not have any pharmacists. As “teaching and research” are a component of a pharmacist’s profession [14], the lack of these professionals in some institutions disappointingly shows that they may not be considered as valued in all of these hospitals.

Encouragingly, from 1359 hospitals labeled as “High Complexity”, 67.4% had pharmacists, and these data stood out when compared to “Medium Complexity” hospitals with only 42.2% having pharmacists. Similarly, there was a predominance of pharmacists in specialized hospitals compared to general hospitals. Yet, some specialized hospitals still lacked access to pharmaceutical care services which may impact on patient-specific successful clinical outcomes [30, 31].

Despite the importance of pharmacists in hospital settings, legally in Brazil, their presence should be guaranteed in oncology and psychiatric hospitals [32, 33]. In our study, more than 80% of the hospitals labeled as “oncology specialized” had pharmacists; however, this may be due to their activities in compounding pharmacy, especially regarding antineoplastic medicines [32], and it is unclear if these pharmacists have further roles that cover more aspects of pharmacy services as outlined in the Basel Statements [7]. In psychiatric hospitals, the Brazilian hospital pharmacy landscape in this study was shown to be inappropriate, lacking professionals properly trained to solve medication-related issues, despite the legal requirements and strict control by sanitary and civil authorities upon these substances [33, 34]. The absence of the health care professional should be highlighted and inspected by sanitary authorities.

Regarding the management of hospitals, most were managed by municipalities. The minority, managed by states, showed a higher prevalence of pharmacists. Apparently, state management agencies present more severe criteria and/or control to organize health care professionals, but more studies are necessary to evaluate the influence of these variables upon pharmacy practice.

As hospitals were clustered by ownership, those labeled as “business entities” had the lowest pharmacist to bed ratios. In contrast, non-profit organizations showed a prevalence of 63.8% of pharmacists. Also, more than half (52.02%) of the hospitals under public administration did not have pharmacists. Thus, many public institutions are currently not legally compliant. On the other hand, non-profit organizations tended to spend more money on human resources, perhaps as they are not aiming for profit. Non-profit organizations have recognizable contributions to improve public health all over the world [35–37], and in the perspective of pharmacist workforce in Brazil, it appears to be an advantage, perhaps indicating that they see the benefit of integrating pharmacists into their multidisciplinary teams.

Additionally, the distribution of pharmacists was uneven across the Brazilian regions. In North, Northeast, and Central-West regions, less than half of hospitals had pharmacists, compared to data from South and Southeast regions. The region with the highest presence of pharmacists and the highest population to hospitals ratio was the Southeast region. In contrast, the Northeast region presented the highest absolute count of hospitals with lowest prevalence of pharmacists. These regional differences are far beyond pharmacist presence. Historically, Brazilian regions present extreme inequalities (demographic, economic, social, cultural, and sanitary) even for access to the health system [38, 39].

In the present study, a positive correlation was obtained between GDP and the total number of hospital pharmacists in each Brazilian state. These results are in accordance with Bates et al. They found a positive correlation between economic development and expenditure on health and pharmacist availability [18]. The south and southeast regions of Brazil make up 76.1% of the Brazilian Gross National Product [22]. It therefore could be plausible that pharmacists are not seen as an essential resource and only considered as an investment when additional resources are available. This contrast between need for services and access to it, in a medium- to long-term perspective, could result in a collapse in the country’s health system, as the population ages [22], and they demand more intensive healthcare services, including pharmaceutical care [40].

While in developed countries such as the USA, pharmacy technicians are taking on administrative and clinical responsibilities to support pharmacists, this is not yet the case in Brazil [12, 15, 16]. Brazilian studies conducted by Magarinos-Torres et al. and Freitas et al. described factors negatively influencing pharmacists’ clinical responsibilities: the hospital manager is not interested in clinical activities or does not recognize the pharmacist as a clinical professional; insufficient clinical training in undergraduate studies; difficulty interacting with other professional categories; pharmacists do not recognize themselves as healthcare professionals and do not understand they can perform clinical functions; no structural contribution to develop clinical activities; administrative, logistic and bureaucratic time-consuming tasks, not leaving time for clinical activities [17, 41].

This study has provided an important snapshot of the pharmacist workforce across Brazilian hospitals. The main limitation of this study is the use of a government-managed database, in a cross-sectional study; thus, some data may be out of date. The mandatory registration of all Brazilian healthcare facilities in CNES was established by law and data are validated by unannounced visits [42]. Moreover, the database was unable to provide information in which field the pharmacist was practicing (clinical or administrative functions).

## Conclusion

Pharmacists are distributed unevenly in Brazil. The pharmaceutical workforce is slightly concentrated in specialized hospitals, with non-profit organization ownership in the Southeast region. However, it was clear there was a lack of pharmaceutical registration in hospital settings, especially in general hospitals located in Northeast, North, and Central-West Brazilian regions. Law enforcement should be performed to ensure all hospitals have, at least, one pharmacist per 50 beds, assuring quality in the healthcare process and more success in clinical outcomes. Finally, further exploratory studies are required to assess the presence of the pharmacist and their work conditions on a local scale, focussing on their clinical and administrative roles and reinforcing their need in hospitals.

## Abbreviations

CNES: National Database of Healthcare Facilities
FIP: International Pharmaceutical Federation
GDP: Gross Domestic Product
IBGE: Brazilian Institute of Geography and Statistics
WHO: World Health Organization
